# Mast Cells: Pivotal Players in Cardiovascular Diseases

**DOI:** 10.2174/157340308785160624

**Published:** 2008-08

**Authors:** Ilze Bot, Theo J.C van Berkel, Erik A.L Biessen

**Affiliations:** 1 Division of Biopharmaceutics, Leiden/Amsterdam Center for Drug Research, Leiden University, Gorlaeus Laboratories, Einsteinweg 55, 2333 CC, Leiden, The Netherlands; 2 Pathology, University of Maastricht, P. Debeyelaan 25, 6202 AZ Maastricht, The Netherlands

**Keywords:** Cardiovascular diseases, atherosclerosis, mast cell, plaque stability, proteases, aneurysm.

## Abstract

The clinical outcome of cardiovascular diseases as myocardial infarction and stroke are generally caused by rupture of an atherosclerotic plaque. However, the actual cause of a plaque to rupture is not yet established. Interestingly, pathology studies have shown an increased presence of the mast cell, an important inflammatory effector cell in allergy and host defense, in (peri)vascular tissue during plaque progression, which may point towards a causal role for mast cells. Very recent data in mouse models show that mast cells and derived mediators indeed can profoundly impact plaque progression, plaque stability and acute cardiovascular syndromes such as vascular aneurysm or myocardial infarction. In this review, we discuss recent evidence on the role of mast cells in the progression of cardiovascular disorders and give insight in the therapeutic potential of modulation of mast cell function in these processes to improve the resilience of a plaque to rupture.

## 1. INTRODUCTION

Already in 1876 during his thesis research Paul Ehrlich discovered a new, highly potent inflammatory cell type and designated it “mast cell”, after the basophilic storage granules [1-3]. Mast cells were found to respond to various exogenous signals from bacteria, viruses and parasites *via* recognition receptors, such as Toll-like receptors and immunoglobulins [4-6]. Mast cells are not only involved in first line defence against pathogens but also represent important mediators in diseases such as allergy and asthma, once they become dysregulated in response to an excess of allergen or allergen-specific IgE [5, 6]. Mast cells are laden with granules containing a wide variety of mediators, such as the proteases chymase, tryptase and the cathepsins, the vasoactive histamine, heparin and a plethora of cytokines such as Tumor Necrosis Factor α (TNFα), chemokines (e.g. Interleukin 8 (IL-8)) and other growth factors (e.g. Vascular Endothelial Growth Factor (VEGF), basic Fibroblast Growth Factor (bFGF)) [6].

Acute cardiovascular syndromes, such as myocardial infarction and vascular aneurysm formation, are still a major cause of death on the Western society. Atherosclerosis, one of the main cardiovascular disorders causing the acute cardiovascular events, is considered a chronic inflammatory disease, involving many inflammatory cells such as macrophages and T-lymphocytes [7, 8]. At later stages, when plaques become clinically manifest, thrombosis, coagulation and fibrinolysis will contribute to the escalation of the disease, which culminates in acute cardiovascular syndromes. Mast cells reside in the perivascular tissue of healthy arteries and during the progression of atherosclerosis, the cells accumulate in the adventitia and the shoulder region of the atherosclerotic plaque. Also the heart is one of the organs rich in mast cells. The whole array of mediators released by mast cells after its activation suggests that they could be involved in the development of atherosclerosis and vascular aneurysm formation, in which immune cells were demonstrated to play a central role. However, it remains to be elucidated whether mast cells are actively modulating the morphology of the arterial wall, thereby being instrumental in atherosclerotic plaque development and progression, aneurysm formation or causing plaque rupture.

In this review, we will outline present knowledge on the contribution of mast cells to cardiovascular diseases in general. Particular emphasis will be on the pathobiologies with an inflammatory component such as the development and progression of atherosclerosis, as well as to myocardial infarction and vascular aneurysm formation. Furthermore, we will discuss *in vitro* and *in vivo* models that have recently given more insight into the mechanisms by which mast cells exert their pathogenic effects. Finally, the therapeutic potential of modulation of mast cell function will be evaluated.

## 2. TECHNIQUES IN MAST CELL RESEARCH

Human pathology studies have provided ample evidence for the presence of a variety of immune and inflammatory cell types in diseased tissue and these studies have been essential in identifying the major cellular players in the progression of disease. Mast cells can easily be detected in tissue sections through a variety of mast cell staining protocols that are available at present. Toluidin Blue as well as a Chloro-Acetate Esterase (CAE) staining both stain the granulae of mast cells [9], while immunohistochemical analysis for CD117, tryptase or chymase can help to conclusively identify mast cell presence in tissues. Mucosal mast cells, present in e.g. lung and colon tissue and connective tissue type mast cells found in tissues like the perivascular tissue can be distinguished by an Alcian Blue/Saphranin O staining [10, 11]. 

Animal models have greatly facilitated to understand the molecular mechanisms involved in cardiovascular diseases, be it atherosclerosis, ischemia/reperfusion injury, or aneurysm formation. In atherosclerosis, e.g. high fat diet fed rabbits and genetically modified hyperlipidemic mice, such as the ApoE^-/-^ [12, 13], LDLr^-/- ^[14] or the ApoE3Leiden transgenic mice [15] have all been demonstrated to develop atherosclerotic plaques in aortic valve area, the aortic arch and carotid arteries and can be used for intervention studies. To induce myocardial infarction, coronary artery ligation models have been developed resulting in ischemia of the heart, often followed by reperfusion to study the inflammatory response [16, 17]. Likewise, a number of models have been described to identify key players in vascular aneurysm formation, which are generally based on elastase perfusion of the aorta [18], peri-aortic chemical injury [19], arterial clamping [20] or Angiotensin II infusion in atherosclerotic ApoE^-/-^ mice [21]. 

Mast cell deficient animals were reported already in 1978, when Kitamura and colleagues first described W/Wv mice that were virtually devoid of tissue and skin mast cells [22]. These mice, besides being anemic, sterile and lack of hair pigmentation, were demonstrated to only have 1% of the normal number of skin mast cells compared to wild-type mice, while in other tissues of these mice mast cells are completely absent. W/Wv mice were found to carry a mutation in the C-Kit gene which results in an intrinsic defect in hematopoietic stem cells and dysregulated hematopoiesis. Injection of these mice with bone marrow from wild-type mice resulted in a repopulation of mast cells. The anemic Sl/Sl^d^ mouse has a comparable phenotype as the W/Wv mouse, however in this strain bone marrow transfer does not rescue the phenotype, suggesting that in these mice environmental factors needed to induce mast cell differentiation and maturation are absent [23, 24]. A mutation of the C-kit gene in the white spotting (Ws) locus resulted in the mast cell deficient Ws/Ws rat [25]. More recently, a new mast cell deficient mouse was described that bears the spontaneously arisen W-sash (W^sh^) inversion mutation. Unlike the previous two strains, mast cell deficiency is not accompanied by anemia and sterility [26]. These Kit(W^-sh^/W^-sh^) mice can also be repopulated with mast cells by injection of either bone marrow cells or cultured bone marrow derived mast cells (BMMCs), rendering this mouse a very interesting animal for studying mast cell biology *in vivo*. 

Another approach in mast cell research, albeit not frequently pursued in cardiovascular disease models, involves systemic or focal mast cell activation in animals. To date, most research has been conducted in disease models of asthma, allergy and arthritis. Mast cells can be activated in various ways, of which IgE stimulation and hapten sensitization/challenge are widely known [27, 28]. Compound 48/80 is a well-known mast cell activator used in *in vitro* as well as *in vivo *studies [29]. In the next sections, human pathology studies as well as the use of *in vitro* and *in vivo* models investigating the role of mast cells and mast cell constituents in cardiovascular disorders will be described.

## 3. MAST CELLS IN ATHEROSCLEROSIS

Already in 1953, Constantinides reported that mast cells might be involved in the susceptibility to experimental atherosclerosis [30]. In this study, the author suggested that the heparin stored in mast cell granules could be atheroprotective. In a follow up study in 1954, Constantinides and coworkers stated that myocardial tissue from patients suffering from atherosclerosis displayed reduced numbers of mast cells per microscopic field compared to younger individuals not suffering from atherosclerosis [31]. As already demonstrated in his first report on experimental atherosclerosis, this phenomenon was again attributed to the atheroprotective role of mast cell derived heparin [30]. In the following decades, the paradigm gradually shifted towards a pro-atherogenic role for mast cells, as mast cell numbers in the intima, media as well as in the perivascular tissue (the adventitia), were in fact found to be increased with the progression of atherosclerosis [32]. 

Mast cell constituents such as histamine, prostaglandin D2 and leukotrienes play a role in atherogenesis and its clinical manifestations by affecting endothelial cell function and vasoreactivity. This hypothesis is strengthened by the recent finding that increased mast cell numbers in the adventitia of coronary arteries associate with vasospasms [33]. Chymase, a mast cell protease, can contribute to vasoconstriction by converting Angiotensin I into the vasoactive Angiotensin II (AngII), independently of Angiotensin-I Converting Enzyme (ACE) [34, 35]. Furthermore, chymase has been demonstrated to convert big endothelin-1 (ET-1) to 31-amino acid-length endothelins, ETs(1-31), which also act vasoconstrictive [36, 37]. 

Although mast cells were on one hand demonstrated to inhibit macrophage induced oxidation of Low Density Lipoprotein (LDL) [38, 39], mast cells have on the other hand been suggested to contribute to atherogenesis through secretion of heparin proteoglycans (HPG) containing granules, which in turn bind LDL. HPG bound LDL can subsequently be captured in the intima by phagocytosing macrophages, leading to enhanced lipid accumulation and lesion progression [40, 41]. Additionally, mast cell chymase is able to degrade apoE and apoAII, leading to a reduction of cholesterol efflux from macrophages to these apolipoproteins, which enhances foam cell formation [42]. Furthermore, oxidized LDL (OxLDL), modified in the intima, can induce mast cell degranulation, resulting in atherosclerotic lesion expansion through a self-amplifying cascade and moreover through increased leukocyte influx [43, 44]. Secretion of chemokines Monocyte Chemotactic Protein-1 (MCP-1) and IL-8 by mast cells will also increase leukocyte influx into the plaque [45]. Mast cell progenitors could be recruited to the plaque as well during plaque progression together with other pro-inflammatory cells such as monocytes and lymphocytes. Haley *et al*. described the presence of CCR3 positive mast cells in plaques of atherosclerotic arteries, while the primary ligand of CCR3, eotaxin, was demonstrated to be highly expressed by activated vascular smooth muscle cells [46], suggesting that the chemokine receptor CCR3 is involved in this process. 

Foci of mast cell accumulation were detected in the intima at later stages of plaque formation, mainly near the shoulder region and in areas of early calcification of the plaque [47-50]. As described above, these mast cells may have been recruited to the plaque by chemokines such as eotaxin and adhere to the plaque surface or to extracellular matrix components such as fibronectin, vitronectin or laminin *via* integrins and adhesion molecules like intercellular adhesion molecule (ICAM)-1 [51]. In addition, mast cells may also be (co-)activated upon binding to extracellular matrix molecules, thereby releasing a variety of cytokines into its environment [52, 53].

Intimal mast cells have been demonstrated to contain tryptase, and approximately 40% of the cells contained chymase as well [50]. Both mast cell proteases were able to activate matrix metalloproteinases (MMPs) leading to degradation of collagen and elastin [54-56], consequently resulting in further destabilization of the plaque. As Kovanen and colleagues demonstrated, increased numbers of activated mast cells have been identified in particular in the adventitia of vulnerable and ruptured lesions in patients with myocardial infarction and more importantly, their number was found to correlate with the incidence of plaque rupture and erosion [57-59]. Both may lead to thrombus formation and eventually to acute coronary syndromes such as myocardial infarction. Mast cell activation has recently been demonstrated to have a potential role in the process of endothelial desquamation by demonstrating that the number of subendothelial mast cells correlated with the number of parietal microthrombi, which may have formed at sites of partial plaque erosion [60, 61].

It is not completely clear whether or not adventitial mast cells are capable of modulating intimal processes and instrumental in plaque rupture, although most evidence points in that direction. Pathology studies so far suggest that adventitial mast cells indeed contribute to plaque destabilization and plaque rupture, as mentioned above by release of mast cell proteases chymase and tryptase that can directly degrade matrix molecules themselves but also by activation of MMPs and by conversion of AngI into the proinflammatory, vasoactive AngII [62]. Secondly, mast cells were reported to induce apoptosis of cardiomyocytes [63], vascular smooth muscle cells [64-66] and endothelial cells [67, 68] *in vitro*, which could translate in reduced plaque stability [69, 70].

A third mechanism through which mast cells can contribute to plaque progression and destabilization is by enhancing neovascularization of the plaque. Indeed, in neovascularized areas of human coronary atheromas mast cells were demonstrated to colocalize with intraplaque microvessels [71]. *In vitro* studies showed endothelial proliferation in response to mitogenic factors. Conceivably, VEGF secreted by mast cells [72] could result in enhanced outgrowth of microvessels. Mast cells containing basic Fibroblast Growth Factor (bFGF), a potent angiogenic factor, were located near microvessels in the intima and adventitia of human coronary artery lesions [73], suggestive of a role of these mast cells in neo-angiogenesis. Furthermore, mast cells may, by releasing the vasoactive agent histamine, contribute to microvessel leakage and intraplaque hemorrhage [71].

Only recently, experimental proof was provided in support of a role of mast cells in atherosclerotic plaque development and progression. Sun *et al*. used the mast cell deficient Kit(W^-sh^/W^-sh^) mice, which had been backcrossed with the atherosclerotic LDLr^-/-^ mouse [74]. Atherogenesis in the mast cell deficient LDLr^-/-^ mice was hampered compared to control LDLr^-/-^ mice. Importantly, lipid deposition, macrophage content and plaque apoptosis were reduced, while collagen content and fibrous cap development were increased in these mice, reflective of a more stable phenotype of mast cell deficient plaques. Repopulation of these mice with wild-type BMMCs completely reversed the beneficial effect of mast cell absence. Adoptive transfer of IL-6^-/-^ or IFN↓^-/-^ BMMCs did however not affect lesion progression, implying that mast cell-derived IL-6 and IFN↓ are the prime factors in mast cell dependent plaque initiation. This elegant study conclusively demonstrates the contribution of mast cells to the progression of atherosclerosis, however left unanswered whether mast cells modulate the atherogenic response focally or alter the immune status of the mice, in particular as intimal mast cells are rather scarce in mouse plaques. 

In a parallel study by us, we have pursued a different strategy to address the direct role of vascular mast cells. We have applied slightly constrictive perivascular silastic collars at the carotid arteries of apoE^-/-^ mice to stimulate flow-induced atherosclerotic plaque formation [75]. After the development of advanced atherosclerotic plaques, we sensitized the mice and subsequently activated perivascular mast cells focally at the lesion site by adventitial application of a pluronic gel containing a hapten to induce mast cell activation. Focal mast cell activation in the perivascular tissue of advanced plaques sharply increased the incidence of intraplaque hemorrhage and macrophage apoptosis, the latter being both mast cell protease and histamine dependent [76]. Surprisingly, microvascular leakage was highly enhanced after mast cell activation, which might be responsible for the increased intraplaque bleeding. Furthermore, mast cell activation resulted in enhanced leukocyte recruitment to the plaque, which was CXCR2 and VLA-4-mediated. Most importantly, treatment with the mast cell stabilizer cromolyn prevented all of the undesirable phenomena elicited by mast cell activation, rendering mast cell stabilization a new therapeutic approach in the prevention of acute coronary syndromes. 

Mast cell function in cardiovascular disease was recently shown to be in part dependent on galectin-3, which is a member of *N*-acetyllactosamine-binding lectin family expressed amongst others by mast cells [77]. Galectin-3^-/-^/apoE^-/-^ showed an impaired atherogenic response as compared to apoE^-/-^ mice, while plaques of galectin-3^-/-^/apoE^-/-^ mice had a lower number of perivascular inflammatory infiltrates and mast cells than those of apoE^-/-^ mice [78].

The triggers that activate mast cells during lesion progression and plaque rupture are still subject to investigation. As described above, mast cells can be activated by modified LDL particles which concurs with the notion that bioactive phospholipid components might activate mast cells [43, 44], while mast cells have also been described to colocalize with nerve endings [58, 59, 79]. Interestingly, mast cells have previously been demonstrated to be activated by various neuropeptides [80, 81] and stress was seen to induce massive histamine release by mast cells [82]. The presence of neurons in the perivascular tissue in the vicinity of mast cells and the plaque renders neuron-mediated mast cell activation a plausible mechanism in cardiovascular diseases.

## 4. MAST CELLS IN AORTIC VALVE STENOSIS

Aortic valve stenosis (AS) shares many features with atherosclerosis, including infiltration of inflammatory cells such as macrophages and T-lymphocytes, accumulation of oxidized lipids and extensive tissue remodeling [83, 84]. Analogous to atherosclerosis, also in the diseased aortic valve area mast cells were demonstrated to be abundantly present, as visualized by CD117 immunoreactivity and toluidin blue staining [85]. The molecular mechanisms of this pathological process still remains to be elucidated. A few years ago, AngII was suggested to contribute to the pathogenesis of aortic valve stenosis [86]. In this study, Angiotensin I Converting Enzyme (ACE) mRNA was demonstrated to be upregulated in the stenotic aortic valve and to co-localize with macrophages. Interestingly, also mRNA of chymase, which, as mentioned above, is an AngII producing enzyme as well, was significantly upregulated in these stenotic valve areas, while also the number of mast cells as well as the degree of degranulation was highly increased. Furthermore, cathepsin G co-localized with mast cells in these diseased regions [87]. Hence, current evidence suggests that mast cells are implicated in valve remodeling and AS progression as well, although more research is necessary to delineate the exact contribution of mast cells to this disease process.

## 5. MAST CELLS AND MYOCARDIAL INFARCTION

Myocardial infarction occurs mainly following rupture of an atherosclerotic plaque, leading to thrombus formation and vessel occlusion. The heart is a mast cell-rich organ and serum IgE levels were found to be significantly higher in the patients with unstable angina and acute myocardial compared to stable angina pectoris and controls, indicating that IgE might play a role in the pathogenesis of unstable angina pectoris and acute myocardial infarction [88]. Whole blood histamine levels from patients with stable coronary artery disease were elevated compared to control patients and was shown to be correlated to isoprostane levels as measure for oxidative stress [89], suggesting that histamine might be a risk factor in coronary events. Serum tryptase levels after myocardial infarction may also be altered, albeit that data are still subject to some controversy. Van Haelst *et al*. did not detect any differences in tryptase levels between patients with acute myocardial infarction, with unstable angina pectoris or controls that did not suffer from ischemic cardiovascular diseases [90]. These data have later been confirmed by Kervinen *et al*. [91]. On the other hand, Filipiak and colleagues found elevated serum tryptase levels in patients suffering from myocardial infarction with an ST-segment depression [92]. In fact tryptase levels were even reported to be an independent predictor for future coronary artery disease along with age [93].

In a guinea pig model of ischemia/reperfusion, histamine was demonstrated to be released after an ischemic period [94]. Mast cell distribution in the heart was investigated after induction of acute myocardial infarction in rats, showing a clear-cut accumulation of mast cells in the infarcted region of the heart [95]. Also in dogs, the number of mast cells was highly increased after induction of ischemia/reperfusion injury while macrophages in the infarcted myocardium were demonstrated to express Stem Cell Factor (SCF), a potent attractant for mast cells. This led the authors to suggest that mast cells are recruited by macrophage derived SCF [96]. Mast cell chymase was shown to be upregulated in the infarcted left ventricle in a hamster model of MI, while also the number of chymase-positive mast cells in that area was significantly higher than in the sham operated group. Chymase release could in turn result in enhanced AngII levels and indeed, treatment with an AngII type 1 receptor antagonist was seen to decrease mortality rates, while an ACE inhibitor was ineffective [97]. Apparently, AngII produced by mast cell derived chymase is involved in the pathophysiologic state of the heart after MI. In support of this hypothesis, treatment of hamsters with specific chymase inhibitors (BCEAB, TEI-E548 and NK3201) improved cardiac function and mortality rate after the induction of MI [98-100]. Furthermore, chymase can also induce cardiomyocyte apoptosis *in vitro* [63] and in swines that underwent coronary microembolization, mast cell activation status in the ischemic area correlated with the percentage of apoptotic cardiomyocytes [101]. Treatment of these animals with the mast cell stabilizer tranilast resulted in a reduction of mast cell activation and cardiomyocyte apoptosis, suggesting that mast cells contribute to cardiomyocyte apoptosis.

In general, mast cells have been demonstrated to accumulate in the heart after MI, while the exact role of mast cells after the MI however has not completely been elucidated. Mast cells might on one hand enhance the injury by the release of matrix-degrading enzymes such as chymase and tryptase and of pro-inflammatory cytokines and chemokines resulting in accumulation of inflammatory leukocytes in the infarcted area. Also, mast cell activation may aggravate the injury by the induction of cardiomyocyte apoptosis. As a rich source of VEGF and bFGF however, mast cells may on the other hand contribute to the wound healing response and collateral formation after ischemia. Further research will contribute to our understanding of the actual role of mast cells in MI.

## 6. MAST CELLS IN AORTIC ANEURYSMS

Aortic aneurysm development is a chronic degenerative condition, which is characterized by weakening and dilation of the aortic wall that results in a life-threatening risk of rupture and is associated with the development of atherosclerosis [102]. The first study to report a causal relation between mast cell density in the aortic wall and aneurysm rupture dates already from 1981 [103], when Faleiro *et al. *showed a more pronounced presence of mast cells in arteries of the circle of Willis of patients that died of subarachnoid hemorrhage (SAH) after aneurysm rupture, although the number of patients included in this study was very limited. Besides a single case study of a patient that died of coronary artery dissection in which the perivascular tissue was demonstrated to contain a large eosinophil and mast cell infiltrate [104], hardly any studies reported on the contribution of mast cells to aneurysm development up to 1999. In that year, Ihara *et al*. [62] conclusively demonstrated that in aneurysmal tissue of human aortas the number of activated chymase and tryptase positive mast cells was significantly higher compared to non-aneurysmal atherosclerotic tissue. Mast cell derived chymase, as mentioned before one of the mast cell proteases, can convert AngI into AngII independent of the classic renin-angiotensin system (RAS). Ang II in turn is a very important factor in the development of aneurysms and indeed, chymase and AngII converting enzyme (ACE) activity were both upregulated in aneurysmal aortas compared to control tissue [105, 106]. These findings were in part confirmed by Ejiri *et al*. [107] who demonstrated that in sections of thoracic aortic aneurysm (TAA) samples, chymase-positive mast cells accumulated in the pro-inflammatory regions of TAA tissue, suggestive of a causal relationship between the number of activated mast cells and aneurysm rupture. Furthermore, protein extracts prepared from aneurysm tissue were demonstrated to activate pro-matrix metalloproteinase-9 (MMP-9) into its active form [108], which was demonstrated to be inhibited by the chymase inhibitor NK3201, thereby defining yet another pathway by which chymase can contribute to aneurysm development. Tsuruda *et al*. [109] found an increased number of mast cells in abdominal aortic aneurysm (AAA) tissue as compared to atherosclerotic aortas without any aneurysmal change. These authors also showed high immunoreactivity of these mast cells for adrenomedullin (AM), which exerts antifibrotic actions in cardiovascular remodeling, thus resulting in a reduced stability of the vessel wall. In conclusion, although the number of human pathology studies on mast cells in aneurysm is limited and the sample sizes of most studies small, all evidence directs towards a causal relationship between mast cell numbers and the incidence of aneurysm formation.

*In vivo* studies on the role of mast cell biology in aneurysm formation have been focussed on the mast cell protease chymase as an AngII producing enzyme. Chymase was indeed demonstrated to be involved in aneurysm development in an animal model where AAA was induced by application of elastase onto the abdominal aorta of hamsters that were treated with the specific chymase inhibitor NK3201 [110]. The aortic diameter in the NK3201-treated group was significantly reduced compared to the control group, indicating that the chymase inhibitor prevented the development of AAA in this animal model. Similarly, NK3201 treatment of dogs in which AAA was induced by injecting elastase into the abdominal aorta, resulted in a reduced ratio of medial and total vessel area compared to controls [111]. Collectively these studies show that chymase inhibition may be a useful therapy for prevention of abdominal aortic aneurysms.

Recently, Sun *et al*. studied the role of mast cells itself rather than a specific mast cell constituent in an animal model of abdominal aortic aneurysm (AAA) formation, in which AAA was induced by elastase perfusion in mast cell deficient Kit(W^-sh^/W^-sh^) mice and syngenic control mice [112]. In this study, the mast cell-deficient mice failed to develop AAA because of reduced aortic expansion and internal elastic lamina degradation, while conversely compound 48/80 induced mast cell activation in wild-type mice resulted even in enhanced AAA growth. Mast cell stabilization with cromolyn diminished AAA formation in wild-type mice and the authors demonstrated that mast cells participate in AAA formation by influencing angiogenesis, vSMC apoptosis, and matrix-degrading protease expression. Reconstitution of the Kit(W^-sh^/W^-sh^) mice with BMMCs from wild-type or TNFα^-/-^ mice caused susceptibility to AAA formation to be recovered while IL-6^-/-^ or IFNγ^-/-^ BMMCs were ineffective.

Similarly, AAA were induced in mast cell-deficient mutant Ws/Ws rats by peri-aortic CaCl_2 _application, which resulted in a decreased diameter of abdominal aorta compared to the aortic diameter of wild-type rats [113]. In the wild-type rats, AAA formation was accompanied by accumulation of mast cells, T-lymphocytes, IFNγ positive cells and by activated MMP-2 and -9, which was inhibited in the mast cell deficient rats and interestingly, these phenomena could be inhibited by treatment with the mast cell stabilizer tranilast.

In conclusion, these studies demonstrate that mast cells may not only participate in AAA pathogenesis by inducing aortic SMC apoptosis and by releasing proteases like chymase and tryptase, but also have a role in the inflammatory response in aneurysm development.

## 7. CONCLUSIONS AND THERAPEUTIC POTENTIAL

Culminating evidence from pathology and experimental studies points to a key role for mast cells not only in a range of immune diseases but also in various cardiovascular disorders. Perivascular as well as intimal mast cells were seen to contribute substantially to the pathogenesis of atherosclerosis and plaque destabilization (Fig. (**1**)). Mast cells promote lipid accumulation, matrix degradation, apoptosis, increase leukocyte influx into the plaque and enhance microvascular leakage, all resulting in a sharply increased risk of intraplaque hemorrhage, plaque destabilization and rupture. Second, mast cells contribute importantly to aneurysm formation by the release of chymase, while also mast cell IL-6 and IFNγ were seen to be involved. A role of mast cells in the response after acute myocardial infarction seems plausible but still awaits experimental support. Intervention studies in cardiovascular disease models so far (Table **1**) have revealed a number of promising leads that target pathogenic mast cells per se (mast cell stabilizers) or mast cell constituents such as chymase, tryptase, histamine, IFNγ and IL6, which may have high therapeutic potential in the prevention of plaque rupture, aneurysm formation and possibly other cardiovascular disorders.

## Figures and Tables

**Fig. (1):**
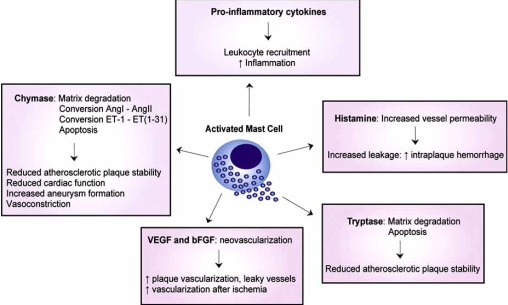
Important mast cell mediators in acute cardiovascular diseases.

**Table 1:** *In Vivo* Intervention Studies Identifying the Role of Mast Cells in Cardiovascular Diseases

Disease Model	Species	Experimental Treatment	Compound/ Gene	Outcome	Reference
Atherosclerosis	Mouse	Mast cell deficiency/adoptive transfer	IL-6^-/-^ or IFNγ^-/-^ BMMCs	↓ Atherosclerotic lesion development	[74]
Atherosclerosis	Mouse	Mast cell stabilization	Cromolyn	↓ IPH incidence	[76]
Myocardial infarction	Hamster	AngII inhibition	AngII type 1 receptor antagonist	↓ mortality rate	[97]
Myocardial infarction	Hamster	Chymase inhibition	BCEAB, TEI-E548, NK3201	↓ mortality rate ↑ cardiac function	[98-100]
Myocardial infarction	Swine	Mast cell stabilization	Tranilast	↓ mast cell activation ↓ cardiomyocyte apoptosis	[101]
AAA (elastase)	Hamster	Chymase inhibition	NK3201	↓ aortic diameter	[110]
AAA (elastase)	Dog	Chymase inhibition	NK3201	↓ aortic diameter	[111]
AAA (elastase)	Mouse	Mast cell deficiency/adoptive transfer	IL-6^-/-^ or IFNγ^-/-^ BMMCs	↓ AAA incidence	[112]
AAA (elastase)	Mouse	Mast cell stabilization	Cromolyn	↓ AAA incidence	[112]
AAA (CaCl_2_)	Rat	Mast cell stabilization	Tranilast	↓ aortic diameter	[113]
